# *Ranunculus sceleratus* as a Model Species to Decrypt the Role of Ethylene in Plant Adaptation to Salinity

**DOI:** 10.3390/plants12020370

**Published:** 2023-01-12

**Authors:** Veronika Prokopoviča, Gederts Ievinsh

**Affiliations:** Department of Plant Physiology, Faculty of Biology, University of Latvia, 1 Jelgavas Str., LV-1004 Riga, Latvia

**Keywords:** 1-methylcyclopropene, air humidity, aquatic species, ethephon, ethylene, growth stimulation, ion accumulation, NaCl, silica xerogel

## Abstract

The aim of the present study was to develop an experimental system for an exploration of ethylene-dependent responses using intact growing *Ranunculus sceleratus* plants and to approbate the system for assessing the role of ethylene in salinity tolerance and ion accumulation. Plants were cultivated in sealed plastic containers in a modified gaseous atmosphere by introducing ethylene or 1-methylcyclopropene (1-MCP), a competitive inhibitor of ethylene action. High humidity inside the containers induced a fast elongation of the leaf petioles of *R. sceleratus*. The effect was ethylene-dependent, as 1-MCP completely blocked it, but exogenous ethylene further promoted petiole elongation. Exogenous ethylene decreased (by 48%) but 1-MCP increased (by 48%) the Na^+^ accumulation in leaf blades of NaCl-treated plants. The experimental system was further calibrated with ethylene and silica xerogel, and the optimum concentrations were found for inducing leaf petiole elongation (10 μL L^–1^ ethylene) and preventing leaf petiole elongation (200 g silica xerogel per 24 L), respectively. The second experiment involved a treatment with NaCl in the presence of 1-MCP, ethylene, or 1-MCP + ethylene, both in normal and high air humidity conditions. In high humidity conditions, NaCl inhibited petiole elongation by 25% and ethylene treatment fully reversed this inhibition and stimulated elongation by 12% in comparison to the response of the control plants. Treatment with 1-MCP fully prevented this ethylene effect. In normal humidity conditions, NaCl inhibited petiole elongation by 20%, which was reversed by ethylene without additional elongation stimulation. However, 1-MCP only partially inhibited the ethylene effect on petiole elongation. In high humidity conditions, ethylene inhibited Na^+^ accumulation in NaCl-treated plants by 14%, but 1-MCP reversed this effect. In conclusion, the stimulation of endogenous ethylene production in *R. sceleratus* plants at a high air humidity or in flooded conditions reverses the inhibitory effect of salinity on plant growth and concomitantly inhibits the accumulation of Na^+^ in tissues. *R. sceleratus* is a highly promising model species for use in studies regarding ethylene-dependent salinity responses and ion accumulation potential involving the manipulation of a gaseous environment.

## 1. Introduction

Studies aimed at understanding the physiological mechanisms of plants adapted to high substrate salinity have gained a special scientific interest within the last few decades, due to the necessity to find out new ways to increase the salinity tolerance of crop plants as well as to understand the functional diversity of wild plant responses to heterogeneous environmental conditions on the background of anthropogenic impacts and climate change [1,2,3]. In contrast to a rather oversimplified but common view that the growth inhibition of plants under high salinity is only due to direct or indirect osmotic and/or toxic effects of NaCl, it is also suggested that hormonal signals are responsible for growth reduction during salinity response [4]. In this respect, it is interesting to note that ethylene has been shown to be involved in growth inhibition in a large number of plant species [5]. Therefore, due to the role of endogenous ethylene in the induction of gene expressions associated with abiotic stress tolerance, it is suggested that ethylene, in cooperation with other hormones, coordinates plant growth with stress responses [6].

So far, only a limited number of plant model systems have been used in studies concerning the role of ethylene in salinity tolerance and the regulation of salt-induced responses. Therefore, the available information is difficult to generalize, especially given the diverse salt resistance mechanisms of halophytic species. It has been suggested that the discrepancy in the role of ethylene in the regulation of salinity responses is due to a lack of comparable studies with diverse plant species over different developmental stages [7]. As a result, clearly contradictory results on the role of ethylene in plant salinity responses can be found in the literature, being described as positive [8,9], neutral [10], or negative [11].

From a point of experimental setup, evidence on the role of ethylene in the regulation of salt-induced responses or salinity tolerance in general can be obtained both indirectly and directly. Indirect evidence is usually based on a relative comparison of intensity and/or time course of ethylene production or 1-aminocylopropane-1-carboxylic acid (ACC) concentration after salt treatment with these of indicators of salinity tolerance, often using different plant species or varieties [11,12]. Another line of evidence on the negative role of ethylene in salinity tolerance is associated with studies using ACC deaminase-producing plant growth-promoting bacteria [13,14]. These results further support the idea that “stress ethylene”, produced in plants as a result of unfavorable environmental conditions, is the main cause of observed growth inhibition. Experimental manipulation of tissue ethylene levels through the application of ethylene-releasing compound chloroethylphosphonic acid (CEPA, ethephon) or ethylene precursor ACC can provide useful information on the possible regulative role of ethylene [8,15]. Relatively solid evidence on the functioning of ethylene reception or the signal transfer system can be obtained using ethylene mutants, either insensitive to ethylene or displaying constitutive ethylene response, but this type of study is mostly restricted to glycophyte model species *Arabidopsis thaliana* [16]. A less commonly used approach in salinity studies is the suppression of endogenous ethylene biosynthesis by inhibitors [10] or the use of ethylene receptor blockers [8]. Surprisingly, experiments with exogenous gaseous ethylene seem to not be used in plant salinity tolerance studies. However, neither ACC nor CEPA can properly replace ethylene treatment in physiological studies [17]. 

Among the available ethylene receptor blockers, 1-methylcyclopropene (MCP) has gained both a theoretical and practical interest. Due to the critical role of ethylene in fruit ripening, MCP has been widely used in fundamental studies regarding the control of fruit ripening [18] and senescence [19], as well as in practical applications related to fruit and vegetable storage [19,20]. The use of MCP in studies involving responses of intact growing plants has been less frequent, but there are several studies involving both foliar spray and gaseous treatment with MCP [21,22,23]. More closely related to the present study, MCP was used for disclosing the hormonal control complexity of hyponastic growth of submerged petioles of *Rumex palustris* [24]. It is evident that the experimental system for assessing the role of endogenous ethylene in salinity responses needs to include both treatments with exogenous ethylene and MCP, as well as their combination, as has been already performed in studies of other ethylene effects with different model species [25,26,27]. 

*Ranunculus sceleratus* L. is an annual semi-aquatic species with a relatively short life cycle, often found in salt-affected coastal habitats [28,29]. The species has been used as a model for studies of ethylene-dependent flooding-induced leaf petiole elongation [30]. Recently, an accession of *R. sceleratus* from a brackish coastal beach habitat was characterized as having a good tolerance to heavy metals and salinity and showed a high shoot metal accumulation capacity [31]. The aim of the present study was to develop an experimental system for the exploration of ethylene-dependent responses using intact growing *R. sceleratus* plants and to approbate the system for assessing the role of ethylene in ion accumulation under saline conditions.

## 2. Materials and Methods

### 2.1. Experimental Setup

A series of experiments were performed with *Ranunculus sceleratus* accession from a coastal seawater-affected habitat in controlled conditions, using plastic containers with modified gaseous atmosphere, in which intact plants growing in soil were placed. Initial experiment (Experiment 1) involved cultivation of *R. sceleratus* plants in vegetative stage in soil at high humidity, resulting from evaporation of water from the plates placed at the bottom of the plants as well as plant transpiration. NaCl was applied to substrate (final amount in substrate 4 g Na^+^ L^–1^), but gaseous atmosphere was modified by MCP and ethylene (5 μL L^–1^), generated by CEPA (Table 1). Plant cultivation in high humidity resulted in elongation of leaf petioles. Further, two-step calibration of the experimental system was performed. First, different amounts of silica xerogel were used to find the amount that reduced humidity so far that petiole elongation response did not appear. This humidity level was designated as “normal humidity”. Second, different concentrations of ethylene in gaseous phase were used in presence of silica xerogel to find efficient range for induction of petiole elongation. Finally, Experiment 2 involved *R. sceleratus* plants induced for flowering and had two different parts, with plants cultivated at high humidity (without silica xerogel) or normal humidity conditions (with silica xerogel). For both parts, plants were treated with NaCl (final amount in substrate 8 g Na^+^ L^–1^), but gaseous atmosphere was modified by MCP and ethylene (10 μL L^–1^), generated by CEPA (Table 1).

### 2.2. Plant Material and Cultivation Conditions

Plants were established from seeds produced in controlled conditions from a seed material initially collected in natural habitat of seawater-affected, wet, and sandy beach in Salacgrīva, Latvia [31]. Detailed procedure for plant establishment and cultivation is described previously [31]. Briefly, seeds were germinated in autoclaved commercial garden soil (Biolan, Eura, Finland) in plastic tissue culture containers for 20 days in a plant growth cabinet, then transplanted to 0.2 L plastic containers filled with mixture of garden soil and quartz sand (Saulkalne S, Saulkalne, Latvia) 1:3 (*v*/*v*), and were adapted to greenhouse conditions: automated greenhouse (HortiMaX, Maasdijk, The Netherlands) with supplemented light that was provided by Master SON-TPIA Green Power CG T 400 W (Philips, Amsterdam, The Netherlands) and Powerstar HQI-BT 400 W/D PRO (Osram, Munich, Germany) lamps (photon flux density of photosynthetically active radiation 380 µmol m^–2^ s^–1^ at the plant level), for a 16 h photoperiod, at a day/night temperature of 22/15 °C, with a relative air humidity of 60–70%. After 2 weeks, plants were transplanted to 0.5 L plastic containers with the same substrate. Plants were fertilized with Yara Tera Kristalon Red and Yara Tera Calcinit fertilizers (Yara International, Oslo, Norway). 

### 2.3. Treatments

For Experiment 1, plants were placed in 48 L containers, with 6 plants per container, and two containers per treatment. The experiment was continued for 7 days. For Experiment 2, plants were placed in 48 L containers for high humidity conditions (6 plants per container and two containers per treatment), and in 24 L container for normal humidity conditions with desiccant (2 plants per container and 3 containers per treatment). The experiment was continued for 6 days.

Treatment with NaCl was performed gradually, applying 1.27 g NaCl dissolved in 100 mL deionized water for each individual container every other day until respective final amount of Na^+^ in substrate was reached. Treatment with NaCl was started on the same day as when plants were placed in closed plastic containers for ethylene and MCP treatment. 

For treatment with ethylene and MCP, intact *R. sceleratus* plants were placed on individual plates in plastic containers that were closed with lids. Commercial formulation Smart Fresh (AgroFresh, Philadelphia, PA, USA), containing cyclodextrin-complexed MCP, was used in the present study for generation of gaseous MCP in plant cultivation containers. Saturating dose of MCP in a gas phase (2 μL L^–1^) was used to prevent the possibility that non-specific binding of MCP in plant tissues affects the specific one [32]. Treatment duration of 12 to 24 h is sufficient to obtain a full response of MCP [33], therefore, to restore CO_2_ concentrations, plant containers were ventilated every 24 h for 1 h and all necessary chemicals were replaced. 

Cerone 480 SL (Bayer CropScience, Leverkusen, Germany), containing 480 g L^–1^ CEPA, was used for production of gaseous ethylene. A stock solution was prepared in ethanol and necessary amount was added to 5 mM Na_2_HPO_3_ buffer and was placed inside a container in a glass vial [17]. For Experiment 1, treatment with ethylene was terminated on day 6, but plants were kept in boxes for additional 24 h, continuing treatment with MCP.

For calibration of experimental system, 24 L containers with 2 plants per container were used, with 3 containers per treatment. For air humidity reduction experiment, plastic plate with different amounts of silica xerogel SGO 50 with indicator (Silcarbon Aktivkohle, Kirchhundem, Germany) was used (0, 100, 200, 300 g). Six plants kept adjacent to boxes on greenhouse bench were used as the controls. Plants were cultivated for 4 days, and silica xerogel was replaced daily after 1 h ventilation of boxes. For ethylene calibration experiment, CEPA was used as described above to obtain concentration of ethylene in containers 1, 10, and 100 μL L^–1^ in presence of 200 g silica xerogel. Additional set of treatments included all concentrations of ethylene as well as control without ethylene in presence of MCP. Plants were cultivated for 6 days, and all necessary chemicals and silica xerogel were replaced daily after 1 h ventilation of boxes. 

### 2.4. Measurements

At the termination of experiments, plant shoots were cut and individually separated in parts (leaf petioles and leaf blades in Experiment 1 as well as in calibration experiments; and leaf petioles, leaf blades, and stems in Experiment 2). Length of petioles was measured. Both fresh and dry mass (after drying at 60 °C for 72 h) of individual plant parts were measured. Water content in tissues was calculated as an amount of water on dry biomass basis. 

Dried plant material was used for measurement of Na^+^ and K^+^ concentration and electrical conductivity (EC) using LAQUAtwin compact meters B-722 (Na^+^) and B-731 (K^+^), and LAQUAtwin conductivity meter B-771 (Horiba, Kyoto, Japan), as described previously [31]. Six individual samples per treatment were independently measured.

### 2.5. Data Analysis

Data were analyzed by KaleidaGraph (v. 5.0, Synergy Software, Reading, PA, USA). Statistical significance of differences for measured parameters between treatments was evaluated by one-way ANOVA using post-hoc analysis by Tukey honestly significant difference test. Significant differences were indicated at *p* < 0.05. Results of ANOVA analysis are presented in Table S1.

## 3. Results

### 3.1. Experiment 1

**Figure 1:**
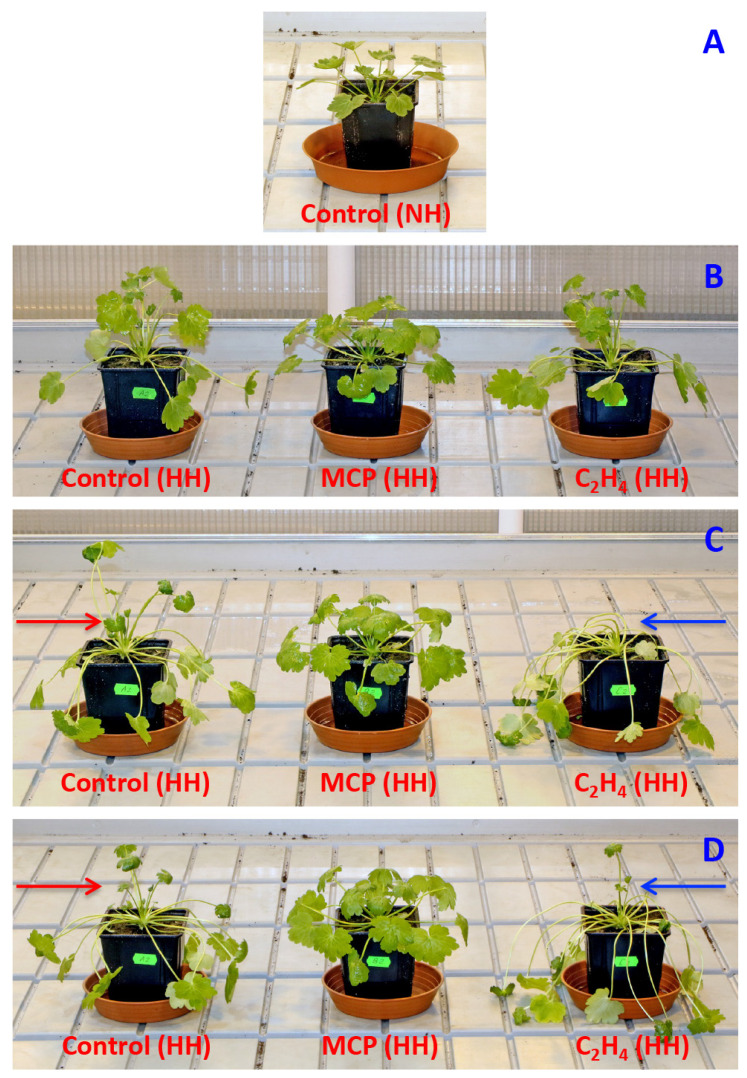
Characteristic morphology of *Ranunculus sceleratus* plants before treatment ((**A**), day 0) and cultivated in closed 48 L containers at high humidity in presence of 1-methylcyclopropene (MCP) and C_2_H_4_. Plants at day 3 (**B**), at day 5 (**C**), and day 7 (**D**). Treatment with C_2_H_4_ was terminated on day 6. NH, normal humidity; HH, high humidity. Red arrows indicate presence of leaves with short petioles in control plants. Blue arrows indicate absence of leaves with short petioles in C_2_H_4_-treated plants (**C**) and appearance of new leaves after termination of the treatment (**D**).

**Figure 2:**
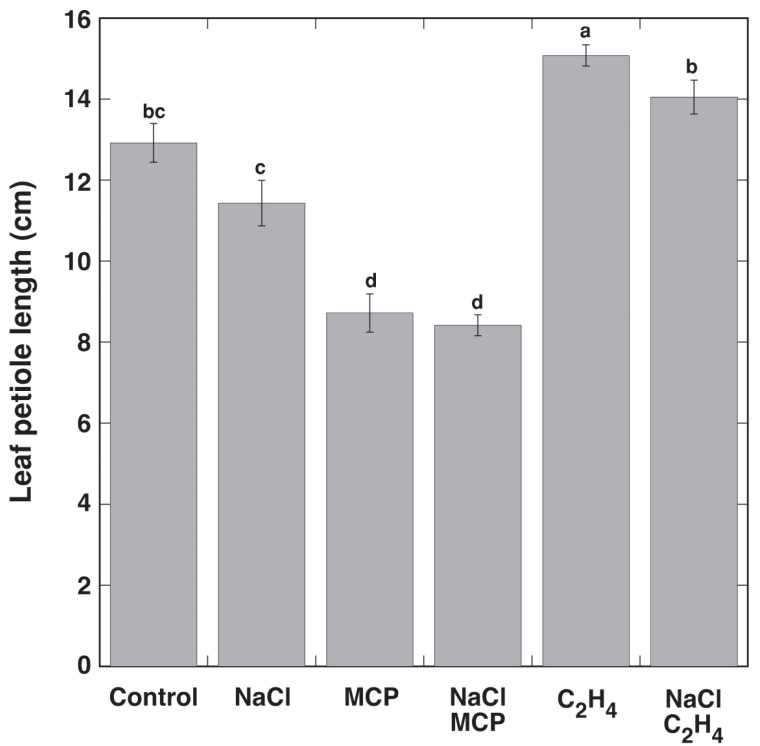
Effect of NaCl, 1-methylcyclopropene (MCP) and C_2_H_4_ treatment on leaf petiole elongation of intact *Ranunculus sceleratus* plants cultivated in closed 48 L containers in high humidity conditions for 7 days. Containers were ventilated for 1 h and new C_2_H_4_ and 1-methylcyclopropene (MCP) releasing solutions were replaced every day. Data are means from six replicates ± SE for each treatment. Different letters indicate statistically significant differences between treatments (*p* < 0.05).

When *R. sceleratus* plants in a vegetative stage were placed in closed containers, increasing the air humidity induced fast elongation of leaf petioles, which was clearly visible already on day 3 (Figure 1B). The presence of ethylene in the container atmosphere further stimulated petiole elongation, but MCP blocked it (Figure 1C). Under the effect of ethylene, all already formed leaves had elongated petioles, and no new leaves appeared (blue arrow, Figure 1C), in contrast to the high humidity control without exogenous ethylene, where leaves with short petioles were still visible (red arrow, Figure 1C). When the ethylene treatment was terminated on day 6, this resulted in the fast formation of new leaves within 24 h (blue arrow, Figure 1D). Measuring the leaf petiole length confirmed that MCP treatment significantly inhibited petiole elongation in high humidity conditions, but treatment with ethylene significantly promoted it (Figure 2). In the conditions of Experiment 1, NaCl treatment did not result in a significant reduction of petiole length.

**Figure 3:**
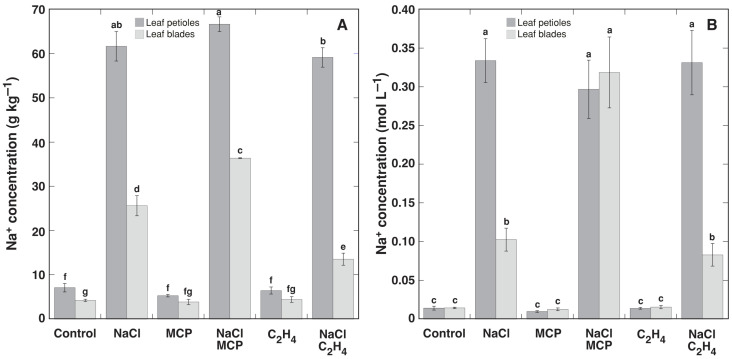
Effect of NaCl, 1-methylcyclopropene (MCP) and C_2_H_4_ treatment on concentration of Na^+^ in leaf petioles and leaf blades of intact *Ranunculus sceleratus* plants cultivated in closed 48 L containers in high humidity conditions for 7 days on dry mass (**A**) and tissue water (**B**) basis. Data are means from six independent measurements ± SE for each treatment. Different letters indicate statistically significant differences between treatments (*p* < 0.05).

**Figure 4:**
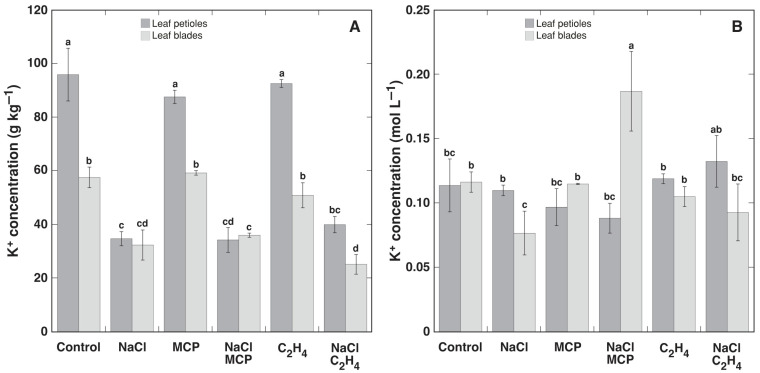
Effect of NaCl, 1-methylcyclopropene (MCP) and C_2_H_4_ treatment on concentration of K^+^ in leaf petioles and leaf blades of intact *Ranunculus sceleratus* plants cultivated in closed 48 L containers in high humidity conditions for 7 days on dry mass (**A**) and tissue water (**B**) basis. Data are means from six independent measurements ± SE for each treatment. Different letters indicate statistically significant differences between treatments (*p* < 0.05).

Treatment with NaCl resulted in the accumulation of Na^+^ in leaves, which was more pronounced in leaf petioles, both on dry biomass and tissue water basis (Figure 3). Treatment with MCP and ethylene did not affect the Na^+^ concentration in leaf petioles. However, treatment with MCP resulted in a significant increase in the leaf blade Na^+^ concentration, but treatment with ethylene reduced the Na^+^ concentration, in comparison to the control plants. The effect of MCP was more pronounced when Na^+^ was calculated on a tissue water basis, but the effect was not significant (Figure 3B). NaCl treatment resulted in a significant reduction of the K^+^ concentration on a dry mass basis in both leaf petioles and leaf blades, but this effect was not significantly affected by additional treatment with MCP or ethylene (Figure 4A). However, when expressed on a tissue water basis, only the K^+^ concentration in leaf blades was significantly decreased by NaCl, and MCP reversed this effect and even stimulated additional K^+^ accumulation (Figure 4B). Ethylene had no significant effect in respect to K^+^ concentration on the tissue water basis.

### 3.2. Calibration of the System

**Figure 5:**
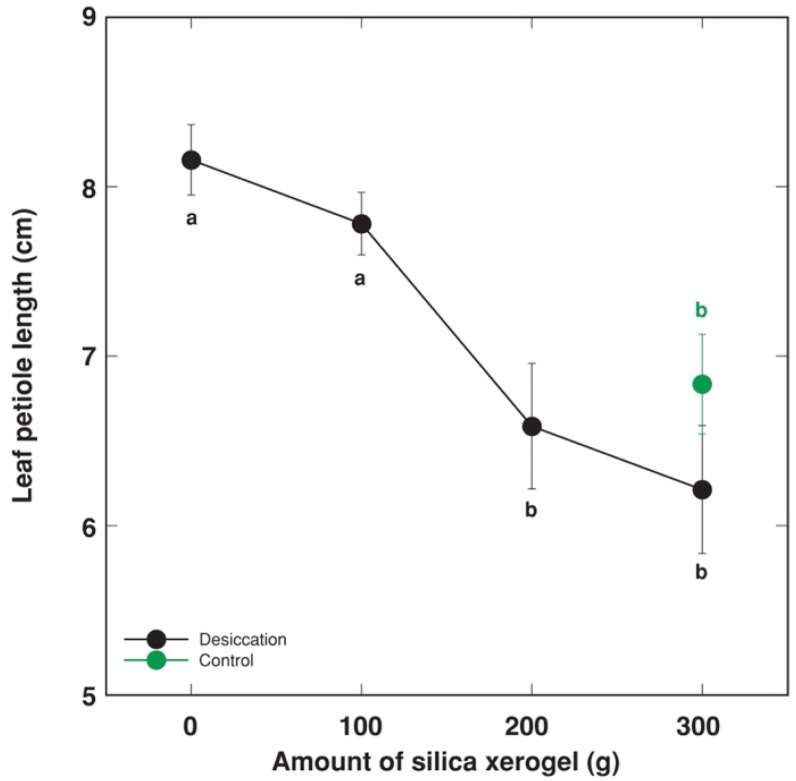
Effect of increasing amount of silica xerogel on leaf petiole elongation of intact *Ranunculus sceleratus* plants cultivated in closed 24 L containers for 4 days. Silica xerogel was replaced each day. Control plants were cultivated outside the containers in greenhouse. Data are means from six replicates ± SE for each treatment. Different letters indicate statistically significant differences between treatments (*p* < 0.05).

Silica xerogel was used as desiccant to decrease the humidity in closed containers with *R. sceleratus* plants. In a 24 L container containing two plants, the daily use of 200 g of fresh silica xerogel was effective enough to prevent the induction of leaf petiole elongation, compared to plants grown at the humidity conditions of the greenhouse (Figure 5). Therefore, the humidity level inside a container in the presence of silica xerogel was designated as the “normal humidity”.

**Figure 6:**
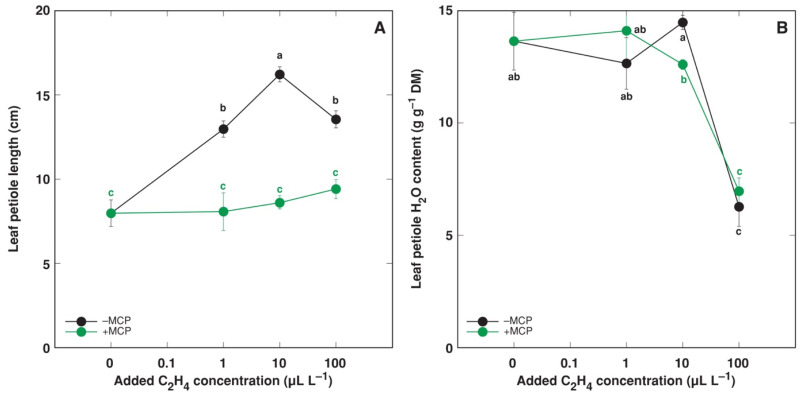
Effect of increasing concentration of C_2_H_4_ (without or with 1-methylcyclopropene, MCP) on leaf petiole elongation (**A**) and leaf petiole water content (**B**) of intact *Ranunculus sceleratus* plants cultivated in closed 24 L containers in normal humidity conditions (with silica xerogel) for 6 days. Containers were ventilated for 1 h and new C_2_H_4_ and 1-methylcyclopropene (MCP) releasing solutions that were replaced every day. Data are means from six replicates ± SE for each treatment. Different letters indicate statistically significant differences between treatments (*p* < 0.05).

Further, *R. sceleratus* plants were cultivated in the presence of 200 g of silica xerogel to obtain a dose-response relationship between the exogenous ethylene concentration and petiole elongation in normal humidity, with and without MCP (Figure 6). The maximum elongation response was evident at 10 μL L^–1^ ethylene in the gas phase, and the response was completely blocked by MCP (Figure 6A). At 100 μL L^–1^ ethylene, the petiole elongation diminished, and this was associated with a significant desiccation of petiole tissues, which was not prevented by MCP treatment (Figure 6B). 

### 3.3. Experiment 2

**Figure 7:**
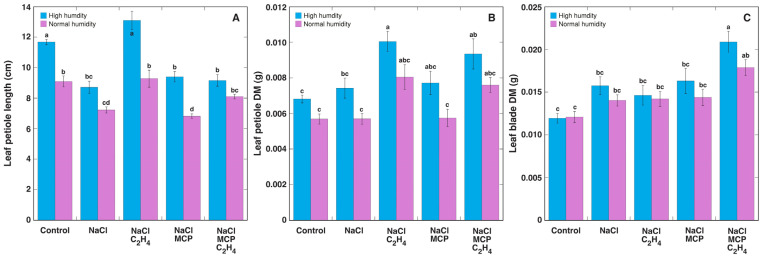
Effect of NaCl, 1-methylcyclopropene (MCP), C_2_H_4_ treatment on elongation of leaf petioles (**A**), leaf petiole dry mass (**B**), and leaf blade dry mass (**C**) of intact *Ranunculus sceleratus* plants cultivated at high humidity and normal humidity. Plants were cultivated in closed 48 L (high humidity) and 24 L (normal humidity with desiccation) containers for 6 days. Containers were ventilated for 1 h and new C_2_H_4_ and 1-methylcyclopropene (MCP) releasing solutions were replaced every day. Data are means from six replicates ± SE for each treatment. Different letters indicate statistically significant differences between treatments (*p* < 0.05).

In Experiment 2, *R. sceleratus* plants were induced to flowering already before treatments, therefore, both the high humidity conditions and ethylene treatment stimulated stem development (Figure S1). Under high humidity, NaCl treatment resulted in the inhibition of leaf petiole elongation growth by 25%, and this effect was reversed by ethylene treatment with no additional growth stimulation (Figure 7A; Table 2). MCP fully prevented this effect of ethylene. Similar effects were evident in normal humidity, but the inhibition of petiole elongation was less pronounced, and MCP only partially prevented the reversible effect of ethylene on petiole length. The dry biomass of petioles was not significantly affected by NaCl, but ethylene treatment resulted in a significant biomass increase by 47%, which was not fully reversed by MCP (Figure 7B; Table 2). In contrast, the leaf blade mass was relatively insensitive to all treatments, and a significant increase of 75% was seen only in NaCl-treated plants in the presence of both ethylene and MCP (Table 2; Figure 7C).

**Figure 8:**
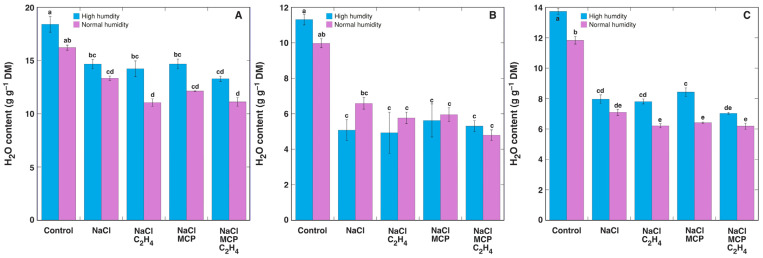
Effect of NaCl, 1-methylcyclopropene (MCP), C_2_H_4_ treatment on water content in leaf petioles (**A**), leaf blades (**B**), and stems (**C**) of intact *Ranunculus sceleratus* plants cultivated at high humidity and normal humidity. Plants were cultivated in closed 48 L (high humidity) and 24 L (normal humidity, with desiccation) containers for 6 days. Containers were ventilated for 1 h and new C_2_H_4_ and 1-methylcyclopropene (MCP) releasing solutions were replaced every day. Data are means from six replicates ± SE for each treatment. Different letters indicate statistically significant differences between treatments (*p* < 0.05). DM, dry mass.

The water content in leaf petioles, leaf blades, and the stems of the control plants was higher in high humidity conditions in comparison to normal humidity conditions (Figure 8). The water content significantly decreased in NaCl-treated plants but was not affected by additional treatment with ethylene or MCP.

**Figure 9:**
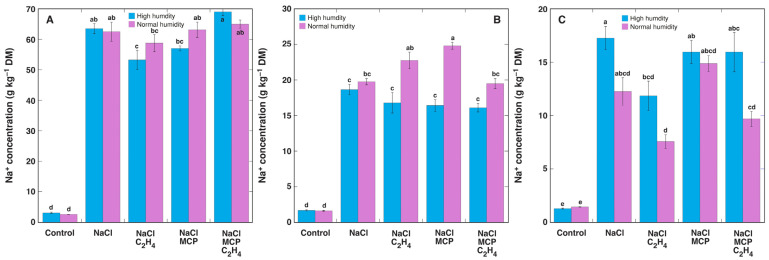
Effect of NaCl, 1-methylcyclopropene (MCP), C_2_H_4_ treatment on Na^+^ concentration in leaf petioles (**A**), leaf blades (**B**), and stems (**C**) of intact *Ranunculus sceleratus* plants cultivated at high humidity and normal humidity. Plants were cultivated in closed 48 L (high humidity) and 24 L (normal humidity, with desiccation) containers for 6 days. Containers were ventilated for 1 h and new C_2_H_4_ and 1-methylcyclopropene (MCP) releasing solutions were replaced every day. Data are means from five independent samples ± SE for each treatment. Different letters indicate statistically significant differences between treatments (*p* < 0.05). DM, dry mass.

The Na^+^ concentration in NaCl-treated plant leaf petioles showed the same type of response as evident for leaf blades in Experiment 1, i.e., ethylene decreased it but MCP together with ethylene resulted in an Na^+^ increase, but it was pronounced only in high humidity conditions (Figure 9A). The Na^+^ concentration in leaf blades of NaCl-treated plants was little affected by the treatment with ethylene or MCP in high humidity conditions (Figure 9B). However, it increased in leaf blades of MCP-treated plants in normal humidity conditions. The most pronounced effect of ethylene on Na^+^ concentration in NaCl-treated plants was evident in newly developing stems (Figure 9C). In high humidity conditions, ethylene significantly decreased Na^+^ accumulation, and MCP restored it. In normal humidity conditions, ethylene also lowered Na^+^ concentration, but this effect was not fully prevented by MCP treatment.

**Figure 10:**
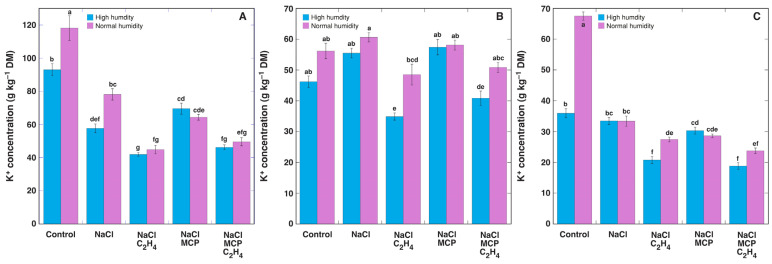
Effect of NaCl, 1-methylcyclopropene (MCP), C_2_H_4_ treatment on K^+^ concentration in leaf petioles (**A**), leaf blades (**B**) and stems (**C**) of intact *Ranunculus sceleratus* plants cultivated at high humidity and normal humidity. Plants were cultivated in closed 48 L (high humidity) and 24 L (normal humidity, with desiccation) containers for 6 days. Containers were ventilated for 1 h and new C_2_H_4_ and 1-methylcyclopropene (MCP) releasing solutions were replaced every day. Data are means from five independent samples ± SE for each treatment. Different letters indicate statistically significant differences between treatments (*p* < 0.05). DM, dry mass.

The K^+^ concentration in leaf petioles, leaf blades, and the stems of control plants was higher in normal humidity conditions in comparison to that in high humidity (Figure 10). NaCl treatment significantly decreased the K^+^ concentration only in leaf petioles and it was further decreased by ethylene, but MCP treatment could not prevent this effect (Figure 10A). However, ethylene treatment also decreased the K^+^ concentration in the leaf blades and stems of NaCl-treated plants, and MCP was also unable to prevent it (Figure 10B,C).

**Figure 11:**
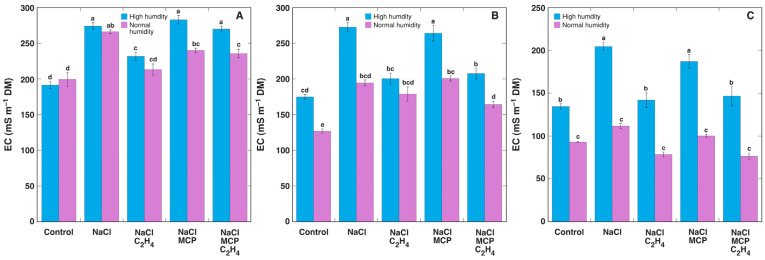
Effect of NaCl, 1-methylcyclopropene (MCP), C_2_H_4_ treatment on electrical conductivity (EC) in leaf petioles (**A**), leaf blades (**B**), and stems (**C**) of intact *Ranunculus sceleratus* plants cultivated at high humidity and normal humidity. Plants were cultivated in closed 48 L (high humidity) and 24 L (normal humidity, with desiccation) containers for 6 days. Containers were ventilated for 1 h and new C_2_H_4_ and 1-methylcyclopropene (MCP) releasing solutions were replaced every day. Data are means from five independent samples ± SE for each treatment. Different letters indicate statistically significant differences between treatments (*p* < 0.05). DM, dry mass.

As shown by the measurement of tissue EC, the total level of electrolytically active substances tended to be higher in high humidity conditions, especially, in leaf blades and stems (Figure 11). NaCl treatment increased EC, and ethylene significantly decreased it; however, MCP could not prevent this effect of ethylene.

## 4. Discussion

Ethylene can have both an inhibitory and stimulatory effect on the growth of plant organs, but the particular effect is species-specific and depends on both endogenous and environmental factors [5]. Ethylene-induced elongation has been most commonly observed in aquatic plants. In contrast to the majority of vascular land plants, where ethylene inhibits elongation of vegetative organs, in aquatic and several wetland plant species ethylene is a promoter of elongation growth [34]. Classical examples include the growth of the stems of deepwater rice [35,36], as well as leaf petioles in different submerged *Rumex* species [37], and flooded *R. sceleratus* [30,38]. Consequently, it can be expected that ethylene will have a positive effect on the growth of semi-aquatic species *R. sceleratus* plants, opposed to an inhibitory effect of NaCl.

Similarly, ethylene has been shown to have both a positive and negative role in plant salinity tolerance [39]. The most similar to the current study have been those using ethephon or ACC. Thus, ethephon treatment did not change the Na^+^ and K^+^ concentration in NaCl-treated *Arabidopsis thaliana* [16]. However, ethephon decreased leaf Na^+^ and Cl^–^ accumulation in *Brassica juncea* plants under salinity, also reducing H_2_O_2_ and thiobarbituric acid-reactive substance concentration [8]. Surprisingly, ethephon completely inhibited NaCl-induced ethylene production in this model system. Exogenous ACC decreased the salt sensitivity in *Arabidopsis thaliana* [15] through reversion in a salt-induced inhibition of expression of several transcription factor genes [9].

In the present study, ethylene reversed NaCl-induced inhibition of leaf petiole elongation in *R. sceleratus*, and this effect was prevented by MCP treatment (Figure 7). Moreover, ethylene efficiently decreased Na^+^ accumulation in the leaf blades of plants in a vegetative stage grown in high humidity conditions and treated with NaCl (Figure 3), as well as decreased Na^+^ level in stems of flowering-induced plants, and this effect was prevented by MCP treatment in high humidity conditions (Figure 9C). This differed from the results in the study with glycophytic *Solanum lycopersicum* and halophytic *Solanum chilense*, where the inhibition of ethylene biosynthesis did not affect Na^+^ accumulation in either species under salinity [40]. However, the inhibition of ethylene biosynthesis decreased K^+^ accumulation in *Solanum chilense*. In this context, the inhibitory effect of exogenous ethylene on Na^+^ accumulation in *R. sceleratus* was not specific, as ethylene decreased also K^+^ concentration in all parts of NaCl-treated plants (Figure 10). However, in contrast to the effect on Na^+^ concentration, which was prevented by MCP treatment, the inhibitory effect of ethylene on K^+^ accumulation was not fully prevented by MCP. An ability to retain adequate K^+^ concentration in salt-stressed plant tissues has been associated with salinity tolerance for glycophytic species [16,41], but Na^+^ can replace K^+^ for osmotic functions in halophytes [31,42,43]. Therefore, ethylene-dependent decreases in the K^+^ concentration could not have any negative consequences in *R. sceleratus*. Moreover, in natural conditions of salt-affected habitats, *R. sceleratus* has been shown to possess characteristics of an EC controlling species, regulating total soluble ion concentration by interrelated changes in Na^+^ and K^+^ concentration [44].

The main problem with the development of the current experimental system for studying ethylene responses using modified gaseous environment was that the cultivation of semi-aquatic species *R. sceleratus* required a relatively high soil moisture, which was further increased by the treatment of plants with NaCl. This resulted in fast elongation of rosette leaf petioles of plants in the vegetative stage and the rapid development of the stems of plants in a flowering-induced state. In order to reduce the possibility that a high humidity differentially affected ethylene-dependent plant responses, it was necessary to decrease the humidity, which was efficiently performed by using desiccant silica xerogel. 

So far, the rapid elongation of leaf petioles of *R. sceleratus* has been shown to only occur in flooded plants as an escape response, allowing leaf blades to reach the water surface [45]. The entrapment of ethylene in plant tissues under water caused by a low rate of gas exchange in water has been suggested as a mechanism responsible for petiole elongation [46] as well as other adaptive responses to waterlogging [47]. In the present study, leaf petiole elongation was solely induced by the increased air humidity through ethylene sensing, suggesting that some other induction mechanism is present. One of possible explanations involves an increase of auxin sensitivity by ethylene in submerged petioles [46]. It is likely that the same mechanism also operates in conditions of high air humidity together with a high humidity-induced increase of ethylene biosynthesis. The last presumption can be assessed experimentally by using inhibitors of ethylene biosynthesis in high humidity conditions, for example, aminoethoxyvinyl glycine [40,48]. In natural conditions, *R. sceleratus* plants are extremely tolerant to flooding, due to the ability to quickly increase the petiole elongation rate as well as constitutively high root porosity [45]. Stimulation of endogenous ethylene production in *R. sceleratus* plants in flooded conditions [30] or even at high air humidity (the present study) reverses the inhibitory effect of salinity on plant growth, and concomitantly inhibits the accumulation of Na^+^ in tissues, thus serving as an important adaptative mechanism to sustain photosynthesis when flooded with seawater.

It is relatively well-established that variations in the air humidity can cause dramatic changes in plant growth, physiology, and morphology, including the biomass accumulation [49,50], cuticular wax composition [51], xylem efficiency [52], root hydraulic properties [53], and stomatal functioning [54] etc. In addition, air humidity can modify plant responses to other environmental and developmental clues [55,56]. There are some studies available on the relationship between salinity tolerance and air humidity. In *Jatropha curcas* plants, a high relative humidity improved the plant growth and K^+^:Na^+^ concentration ratio in salinity conditions [57]. Similar results were obtained in tissue culture of *Solanum tuberosum*, showing that plant survival in highly saline conditions significantly increased with a high air humidity, and it was associated with a low Na^+^ accumulation capacity [58]. On the other hand, high relative humidity-induced problems of stomatal functioning in leaves were improved by moderate salinity treatment in *Rosa* × *hybrida* plants [59]. In light of the results from the present study, it is possible that high air humidity increases ethylene biosynthesis or plant sensitivity in response to ethylene, leading to an increased salinity tolerance, in part, due to a lower accumulation of Na^+^.

It is well-known that the physiological status and developmental stage of plants can greatly affect the results in experiments [60]. As a particular example, sensitivity to salinity is usually higher in younger plants, especially, at the seedling stage [61]. It can be concluded that ethylene responses in *R. sceleratus* changed with the developmental stages of plants used in a particular experiment in the present study. In Experiment 1, plants remained in a vegetative stage for the whole experiment. In Experiment 2, both high humidity as well as exogenous ethylene promoted the development of generative structures. It has already been shown earlier that both the submergence and ethylene treatment of flowering-competent *R. sceleratus* plants accelerates the development of flower-bearing structures, stems [62]. Therefore, the effects of ethylene/MCP in the present study also differed between plants in a vegetative state (Experiment 1) and those already forming generative structures (Experiment 2). In particular, after entering a generative phase, rosette leaf petioles became less responsive to high air humidity and ethylene, in respect to elongation growth stimulation. Possible developmental stage-dependence of ethylene and MCP effects clearly needs to be considered in future research. Moreover, special attention should be paid to the specificity of the effects of CEPA-generated ethylene as well as MCP application.

Previously, it was shown that 100 μL L^–1^ ethylene fully saturated the elongation response of isolated *R. sceleratus* petioles [30]. Ethylene released from CEPA inside the cultivation container was used in the present study, and it seems that at 100 μL L^–1^ ethylene, side effects of CEPA breakdown products appeared, as petiole elongation was not further stimulated and leaf petiole water content decreased independently on ethylene binding to the receptors, as MCP did not reverse this response (Figure 6B). CEPA-containing products have indeed been used in agricultural practice, both as defoliants and desiccants [63,64]. Upon breakdown, CEPA also releases phosphoric acid and Cl^–^ [65], which could have an additional effect at a high concentration of CEPA. Therefore, 10 μL L^–1^ ethylene was used in the final experiment.

While MCP can “protect” plants from both endogenous and exogenous ethylene [33], the effect of MCP can also be assessed ambiguously [66]. There are no known additional primary effects of MCP in plants, besides acting as an irreversible inhibitor of ethylene action, but it has been suggested that MCP can potentially have additional biological effects related to the modification of downstream gene expression, leading to an altered metabolism [67], especially, in a long-term situation [68]. Whereas in the present study, treatment with MCP was relatively long (6–7 days), and some potentially ethylene-dependent effects were not fully reversed by MCP treatment, for example, the decrease of the K^+^ concentration and EC (Figure 11). 

## 5. Conclusions

The stimulation of endogenous ethylene production in *R. sceleratus* plants at a high air humidity or in flooded conditions reverses the inhibitory effect of salinity on plant growth and concomitantly inhibits the accumulation of Na^+^ and K^+^ in plant tissues. It appears that *R. sceleratus* could be a promising model species for use in studies of ethylene-dependent salinity responses and ion accumulation potential, involving the manipulation of a gaseous environment. Due to its high tolerance and accumulation potential for heavy metals, it can also be used in studies of putative ethylene-dependent mechanisms of metal tolerance. However, further improvement of the experimental system is necessary, allowing for a more precise control of the air humidity in closed environment. The system should also be supplemented with the use of ethylene biosynthesis inhibitors, which is more limited in time compared to the use of gaseous ethylene and MCP. Non-aquatic salinity-tolerant coastal species are possibly less tolerant to an increased air humidity are also prospective models for research in this model system.

## Figures and Tables

**Table 1:** Experiments and treatments performed with *Ranunculus sceleratus* plants.

Experiment/Treatment	NaCl	MCP	CEPA	Silica Xerogel	Container Volume (L)	Replicates (Boxes/ Plants)
Experiment 1 (high humidity)						
Control	−	−	−	−	48	2/6
NaCl	+	−	−	−	48	2/6
MCP	−	+	−	−	48	2/6
NaCl MCP	+	+	−	−	48	2/6
C_2_H_4_	−	−	+	−	48	2/6
NaCl C_2_H_4_	+	−	+	−	48	2/6
Experiment 2 (high humidity)						
Control	−	−	−	−	48	2/6
NaCl	+	−	−	−	48	2/6
NaCl C_2_H_4_	+	−	+	−	48	2/6
NaCl MCP	+	+	−	−	48	2/6
NaCl MCP C_2_H_4_	+	+	+	−	48	2/6
Experiment 2 (normal humidity)						
Control	−	−	−	+	24	3/6
NaCl	+	−	−	+	24	3/6
NaCl C_2_H_4_	+	−	+	+	24	3/6
NaCl MCP	+	+	−	+	24	3/6
NaCl MCP C_2_H_4_	+	+	+	+	24	3/6

MCP, 1-methylcyclopropene; CEPA, chloroethylphosphonic acid.

**Table 2:** Relative effect of treatments in Experiment 2.

Treatment	High Humidity		Normal Humidity	
	In Respect to Control (%)	Statistical Significance (*p*)	In Respect to Control (%)	Statistical Significance (*p*)
Leaf petiole length				
NaCl	75	<0.05	80	<0.05
NaCl C_2_H_4_	112	ns	102	ns
NaCl MCP	81	<0.05	75	<0.05
NaCl MCP C_2_H_4_	78	<0.05	89	ns
Leaf petiole dry mass				
NaCl	109	ns	100	ns
NaCl C_2_H_4_	147	<0.05	141	ns
NaCl MCP	113	ns	101	ns
NaCl MCP C_2_H_4_	137	<0.05	134	ns
Leaf blade dry mass				
NaCl	132	ns	116	ns
NaCl C_2_H_4_	123	ns	117	ns
NaCl MCP	137	ns	119	ns
NaCl MCP C_2_H_4_	175	<0.05	149	<0.05

ns, not significant.

## Data Availability

All data reported here are available from the authors upon request.

## Supplementary Materials

The following supporting information can be downloaded at: https://www.mdpi.com/article/10.3390/plants12020370/s1. Table S1: results of ANOVA analysis of experimental data; Figure S1: characteristic morphology of *Ranunculus sceleratus* plants cultivated at high humidity (A) and normal humidity (B).
