# Supplementary material for: Ranunculus sceleratus as a Model Species to Decrypt the Role of Ethylene in Plant Adaptation to Salinity
Source: Plants (Basel). 2023 Jan 12;12(2):370. doi: 10.3390/plants12020370 (PMC9862674; doi:10.3390/plants12020370)
Supplement: Supplementary file 1 [file plants-12-00370-s001.zip › plants-2086457-supplementary.pdf]

Article

Ranunculus sceleratus as a Model Species to Decrypt the Role of Ethylene in Plant Adaptation to Salinity

Veronika Prokopoviča and Gederts Ievinsh

Supplementary Table S1. Results of ANOVA analysis of experimental data.

**Experiment**	**Parameter**	***DF***	***F***	***p***
Experiment 1	Petiole length	5	42.6	< 0.0001
	Na^+ concentration on dry mass basis	11	265.4	< 0.0001
	Na^+ concentration on dry water basis	11	38.6	< 0.0001
	K^+ concentration on dry mass basis	11	34.0	< 0.0001
	K^+ concentration on dry water basis	11	3.1	0.011
Calibration of the experimental system	Petiole length (silica xerogel)	4	5.0	0.004
	Petiole length (ethylene)	6	57.4	< 0.0001
	Petiole water content (ethylene)	6	68.8	< 0.0001
Experiment 2	Petiole lenght	9	26.0	< 0.0001
	Petiole dry mass	9	6.1	< 0.0001
	Blade dry mass	9	5.8	< 0.0001
	Petiole water content	9	17.8	< 0.0001
	Blade water content	9	10.8	< 0.0001
	Stem water content	9	122.7	< 0.0001
	Na^+ concentration in petiole	9	162.6	< 0.0001
	Na^+ concentration in blade	9	96.2	< 0.0001
	Na^+ concentration in stem	9	28.7	< 0.0001
	K^+ concentration in petiole	9	53.7	< 0.0001
	K^+ concentration in blade	9	16.7	< 0.0001
	K^+ concentration in stem	9	125.3	< 0.0001
	Electrical conductivity in petiole	9	33.3	< 0.0001
	Electrical conductivity in blade	9	40.4	< 0.0001
	Electrical conductivity in stem	9	47.2	< 0.0001

Suppelemntary Figure S1. Characteristic morphology of *Ranunculus sceleratus* plants cultivated at high humidity (A) and normal humidity (B). Plants were cultivated in closed 48 L (high humidity) and 24 L (normal humidity, with desiccation) containers for 6 days. Containers were ventilated for 1 h and new C_2H_4 and 1-methylcyclopropene (MCP) releasing solutions were replaced every day.

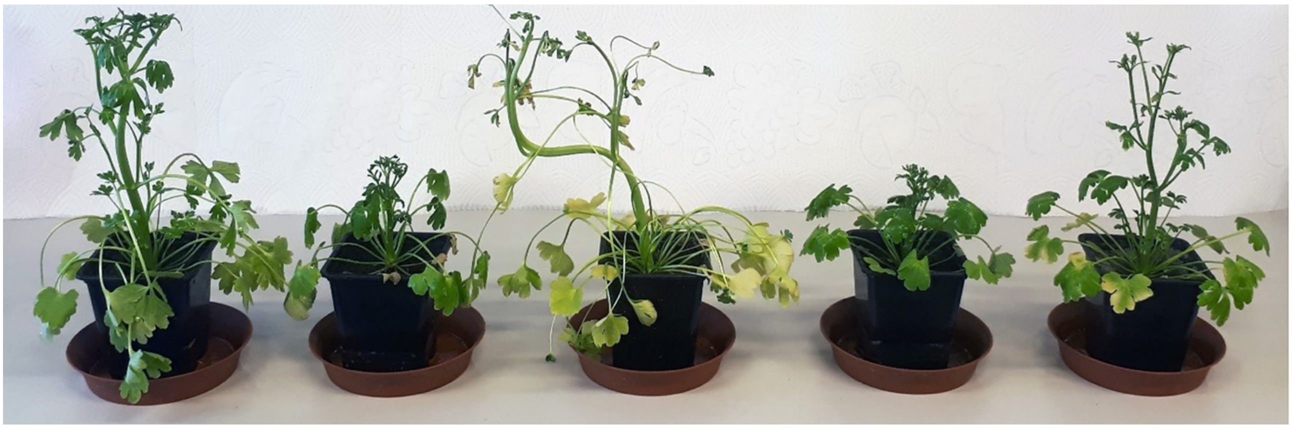

A, at high humidity. From left to right: control, NaCl, NaCl + C_2H_4, NaCl + MCP, NaCl + C_2H_4 + MCP.

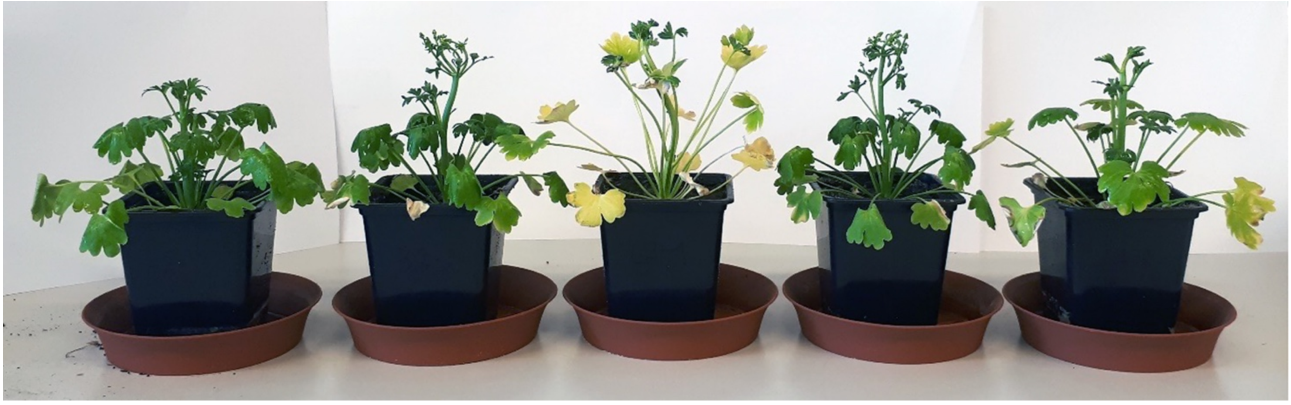

B, at normal humidity. From left to right: control, NaCl, NaCl + C_2H_4, NaCl + MCP, NaCl + C_2H_4 + MCP.
